# Developing and evaluating a proof-of-concept patient safety training programme for health workers in North Macedonia

**DOI:** 10.1136/bmjoq-2025-003473

**Published:** 2025-09-22

**Authors:** Elizabeta Zisovska, Válter R Fonseca, Margarita Spasenovska, Robert Velickovski, Damir Ivanković, Joao Breda

**Affiliations:** 1 University Clinic for Gynaecology and Obstetrics, Skopje, North Macedonia; 2 WHO Country Office, Skopje, North Macedonia; 3 WHO Office on Quality of Care and Patient Safety, World Health Organization Regional Office for Europe, Athens, Greece; 4 Ministry of Health, Skopje, North Macedonia

**Keywords:** Patient safety, Education, Safety culture, Safety Management

## Abstract

**Background:**

Patient safety is a global health priority, yet formal training in patient safety principles for healthcare workers remains limited in many countries, particularly in low-resource or transitional health systems. Similar to other countries in South-Eastern Europe, North Macedonia faces patient safety challenges—including a prevailing blame culture and gaps in standard safety practices. We designed, delivered and evaluated a context-tailored patient safety training programme for healthcare workers in North Macedonia and assessed its impact on participants’ immediate knowledge gains of key patient safety topics.

**Methods:**

A 4-day interactive training workshop was developed following a situational analysis of national safety gaps. Day 1 included awareness-raising sessions for institutional managers. Eighty-five healthcare workers, physicians, nurses and midwives, participated in a 3-day workshop that followed. The curriculum covered priority patient safety domains, such as incident reporting, infection prevention, medication safety and surgical and obstetric safety, delivered through lectures and case-based group exercises. Immediate knowledge gains were measured using a 20-item multiple-choice test administered pretraining and post-training. Pre-training and post-training scores were analysed and compared.

**Results:**

Baseline knowledge was suboptimal with a mean pretest score of 37% of correct answers. Immediately after the training, overall knowledge improved markedly. The mean post-test score reached 72%, a gain of 35 percentage points. All topic areas showed significant knowledge gains. Large improvements were observed in domains with the lowest baseline scores—mean correct responses in surgical safety domain increased from 19% to 76%, in obstetric safety from 18% to 67% and in infection control domain from 31% to 87%.

**Conclusions:**

This proof-of-concept quality improvement research initiative suggests that focused educational interventions could help address patient safety knowledge gaps. Sustaining and expanding such training, by integrating it into routine workforce development and licensing, may help strengthen patient safety culture and practices in similar settings.

**WHAT IS ALREADY KNOWN ON THIS TOPIC**

**WHAT THIS STUDY ADDS**

- A short, context-tailored training programme significantly improved healthcare workers’ immediate knowledge across multiple safety domains, especially in areas with low baseline knowledge such as surgical and infection safety.

**HOW MIGHT THIS STUDY AFFECT RESEARCH, PRACTICE OR POLICY**

- This proof-of-concept quality improvement research initiative supports scaling up participatory, context-specific training as part of continuing professional development. Such training can help foster an enabling environment for peer learning, promote leadership engagement and strengthen safety culture in similar settings.

## Introduction

 Every year, millions of patients suffer harm or death due to unsafe and poor-quality healthcare. Nearly 1 in 10 patients is harmed while being provided care and more than 3 million deaths occur due to unsafe care each year.^1^ The WHO has recognised patient safety as an urgent global health priority and is at the forefront of promoting the link between education and safer care. This led to the development of Patient Safety Curriculum Guide in 2011, launch of the World Health Assembly’s resolution on ‘Global Action on Patient Safety’ in 2019 and publication of the WHO Global Patient Safety Action Plan 2021–2030.^2^,^4^ All of these emphasise health workforce education and training as a strategic objective. This requires system-wide efforts, clear policies, committed leadership, capacity-building and robust data for monitoring improvement at all levels.^5^ Strengthening ‘safety culture’—where errors lead to learning rather than blame—is particularly important in settings where fear of blame has historically impeded reporting and learning from safety incidents.^6 7^

In line with these global priorities, and similar to other countries in Southeast Europe (SEE), North Macedonia continues to face challenges in patient safety and quality of care. A rapid situational analysis conducted in 2023 by our research team identified a number of systemic barriers in North Macedonia: (1) existing individual and organisational blame culture; (2) absence of a systematic patient safety incident reporting and learning system at both facility and national levels; (3) insufficient implementation of standard protocols for various care processes (eg, falls prevention, safe surgery, obstetric safety measures, blood transfusion safety); (4) lack of standardised protocols for clinical communication and care transitions; (5) low compliance with infection prevention and control guidelines and (6) inadequate routine data collection and monitoring of patient safety indicators. These gaps undermine efforts to systematically strengthen patient safety in North Macedonia and point to the need for both knowledge-building and broader culture change.

Progress has been made in recent years: a national commission on healthcare-associated infection control has been established, a national antimicrobial resistance strategy was implemented and the health sector has undergone digitalisation, including the adoption of a national electronic prescription system.^8^ In September 2024, the Government of North Macedonia adopted a new ‘National plan for patient safety 2024–2030’, aligned with the national health strategy.^9 10^ Supported by WHO, this plan promotes a systems approach to safety and includes training and capacity-building as core components. Still, significant challenges remain, as much of the existing workforce lacks formal exposure to patient safety concepts, and there are few mechanisms to cultivate institutional leaders or quality champions. Antibiotic consumption in North Macedonia is high, almost double the European Union average, indicating ongoing issues with infection control and antibiotic stewardship.^11^ In 2014, the Ministry of Health (MoH) created the Agency for Quality and Accreditation of Health Care Institutions as an independent, arm’s-length body mandated with developing quality standards, assessing service quality and accrediting healthcare facilities. The national accreditation process is yet to become fully operational, while assessment of care quality and patient safety is hampered by the lack of systematic data collection on key indicators at both national and facility levels.^12^

Addressing social, political, economic and organisational factors that impact care quality and patient safety begins with strong political commitment and leadership support that involves health system managers, frontline providers, communities and patients.^13^ In North Macedonia, the MoH’s recognition of patient safety as a policy priority has led to a systems approach with support from WHO, including capacity-building initiatives focused on leadership and patient safety culture.

Integration of patient safety topics into health professional education remains limited in many settings despite its recognised relevance. Globally, only a fifth of countries have incorporated patient safety topics into undergraduate and postgraduate training curricula, and roughly one-quarter offer specialised in-service patient safety training for health workers. In the WHO European Region, it is estimated that only about 25% of countries include patient safety in formal, medical and nursing, education programmes for healthcare workers. While patient safety is increasingly being introduced in preservice education, many active health professionals have not received comprehensive training on these concepts.^1^ There are also relatively few studies evaluating patient safety training programmes for healthcare workers, a concerning gap given their importance. The limited available evidence shows that well-designed training programmes can improve patient safety knowledge, practices and outcomes.^14 15^ This indicates a clear need to design, deliver and evaluate context-specific patient safety training interventions for the existing health workforce.

Given the challenges and gaps identified in North Macedonia—and reflecting global calls to action—a tailored patient safety training programme was designed to enhance patient safety competencies—encompassing both foundational knowledge and the ability to apply safety principles in practice. While theoretical knowledge of patient safety principles is important, addressing cultural barriers requires more than traditional or online learning formats. In contexts where discussing safety issues may still be sensitive, participatory approaches can help create an enabling environment for engagement, problem-solving and leadership development.^2 14^ This was a key consideration in designing the intervention presented here. This paper (1) describes the design and delivery of the training programme, informed by the situational analysis, and (2) presents participants’ pretraining and post-training programme knowledge assessment results across key patient safety domains.

## Methods

### Training programme development and delivery

This study is best understood as a quality improvement research initiative, with both implementation and evaluation components. Desk research and semistructured interviews with conveniently sampled key national health system stakeholders were used to conduct an internal rapid situational analysis aimed at identifying specific patient safety needs and challenges in North Macedonia. Findings flagged underperforming areas, to which special attention was paid during the development of the training programme. A summary of these findings is included in the Introduction section, to contextualise the intervention.

Design of the training curriculum was informed by these results and structured around four key objectives: (1) raise awareness and understanding of evidence-based patient safety measures, especially those related to critical processes of care identified as problematic in the situational analysis, such as surgical safety, infection control and obstetric safety; (2) develop skills that foster a positive patient safety culture, including leadership commitment, effective communication and teamwork behaviours; (3) train participants in specific patient safety improvement tools and methods, such as incident reporting systems, root cause analysis and feedback mechanisms for continuous improvement and (4) increase knowledge of the epidemiological and economic burden of unsafe care, underscoring the importance of patient safety for health outcomes and health system sustainability.

The programme targeted two primary audiences: hospital managers and clinical staff, members of quality committees within healthcare organisations. Participants were identified through a call issued by the MoH to healthcare institutions nationwide that were invited to nominate candidates based on their roles in clinical care, management or quality improvement. This process was intended to ensure diversity of professions and representation from key hospitals and clinics, while acknowledging the possibility of selection bias due to varying degrees of interest or institutional engagement.

The first day of training, aimed at managers, served as an awareness-raising session rather than a skill-based training. It introduced core patient safety concepts and explained the objectives and structure of the full training programme. Managers did not complete a knowledge assessment, as the session was not designed to measure educational impact but rather to build leadership engagement and managerial buy-in.

The content and structure of the training programme (online supplemental file 1) drew from existing WHO patient safety improvement resources and tools, including the WHO Patient Safety Curriculum Guide.^2^ We adopted an educational approach that combined expert lectures with interactive, case-based group work. This design was chosen to balance the delivery of core knowledge, combining presentations by international and national patient safety experts with practical skills development through group discussions on real-world case scenarios and problem-solving exercises (online supplemental file 1).

The training was codeveloped and implemented by a team comprising WHO Regional Office for Europe technical staff, WHO Country Office in North Macedonia representatives, and national experts in the fields of patient safety, healthcare quality, infection prevention and control and clinical governance. Programme developers, trainers and facilitators brought experience in both international standards and local implementation challenges.

### Evaluation of training impact

To evaluate the impact of the training on participants’ patient safety knowledge, we developed a questionnaire administered as pretraining and post-training tests. The assessment consisted of 20 multiple-choice questions covering key patient safety domains addressed in the workshop: general patient safety concepts, incident reporting, infection prevention and control, medication and blood transfusion safety, surgical safety and safe childbirth practices, patient identification and continuity of care, communication in healthcare and prevention of adverse events, such as patient falls and pressure ulcers (online supplemental file 1). Questions were aligned with the learning objectives and the Rasch analysis was utilised to calibrate question difficulty and ensure the reliability and validity of the instrument.^16^ Draft questions were reviewed by patient safety experts for content validity and then pilot-tested, outside the study participants, to refine wording and difficulty. No major issues, such as ambiguous or too difficult or easy questions, were identified.

Participants completed the test at two time points—on the first day of the workshop, before any training content was delivered and at the end of the final day of the workshop. Participation in the test was voluntary and anonymous, though strongly encouraged. Coding system matched pretraining and post-training test results for each participant while preserving anonymity in the analysis. Although testing was conducted in the same session block, a small number of participants did not complete the post-test, due to leaving early and choosing not to participate. These individuals were excluded from the analysis of knowledge gain.

### Data analysis

All completed assessment sheets were collected and scored by the study team. For each participant, a total number of correct answers pretraining and post-training were calculated as well as the percentage score. We then computed summary statistics for the pretraining and post-training scores overall, and for each thematic domain of questions. Differences between pretraining and post-training scores were analysed using a paired two-tailed Student’s t-test, appropriate for comparing related samples with each participant serving as their own control. Only participants with both pretraining and post-training test data were included in the analysis of knowledge gain. Participants lost to follow-up were excluded from paired statistical comparisons. No major violations of normality or extreme outliers were observed. Statistical analysis was performed using IBM SPSS Statistics (V.21.0). A significance level of p<0.05 was considered indicative of a statistically significant improvement in knowledge.

## Results

### Training programme delivery and participants

The training was delivered over 4 days in November 2023. The first day was dedicated to participants in health management positions, including hospital directors, department heads, primary care clinic managers, with sessions focusing on leadership, governance and creating a safety culture at the organisational level. The 1-day manager session was not included in the pretraining and post-training evaluation, as it did not cover the full training curriculum and was intended to raise awareness and secure managerial support for the clinical training days. The remaining 3 days were attended by a broader group of healthcare workers, including physicians, nurses, midwives and other clinical staff and covered frontline patient safety practices and skills. Lectures were interwoven with small-group activities. During group work, guided by facilitators, participants analysed and discussed patient safety case scenarios relevant to the local context. Examples included a medication error case, a surgical checklist adherence scenario and a healthcare-associated infection case (online supplemental file 2). The format encouraged participants to share their own experiences, learn from peers and apply theoretical concepts to practice. All training materials, such as presentations, case studies and assessment tools, were provided in Macedonian. Simultaneous interpretation to English was offered for international faculty and facilitators.

A total of 85 healthcare professionals attended the training workshop. Participants represented 14 public institutions, with the large majority (78/85; 91.8%) working in secondary or tertiary care institutions (university clinics and hospitals), and some working in primary healthcare (5/85; 5.9%) and in other institutions (2/85; 2.3%). Almost half of participants were nurses (40/85; 47.1%), more than a third were physicians (33/85; 38.8%) and five were midwives (5.9%). 58 women and 27 men participated, aligning with the gender distribution of North Macedonia’s healthcare workforce.^17^,^19^

Three participants did not complete the post-training knowledge assessment and were considered lost to follow-up. These cases were excluded from paired comparisons, in line with the analysis plan. Reasons included early departure and opting out of the post-test, which could have introduced a minor self-selection bias, as noted in study limitations.

### Pretraining and post-training knowledge evaluation

The baseline test results confirmed knowledge gaps in specific patient safety domains (table 1). The overall pretraining mean score was low—participants on average answered only 37% of the questions correctly. Domains with lowest baseline knowledge were surgical safety practices and safe obstetric practices, with less than 20% of correct answers per domain. It is important to note that these two domains were assessed using only a single test item for each, limiting the reliability of domain-specific interpretation. Other areas with low baseline results included infection prevention and control, patient identification, communication and safe handover practices, and prevention of adverse events. Participants showed better baseline knowledge in general patient safety questions—covering fundamental concepts such as the definition of an adverse event and the idea of a no-blame reporting system—and in medication safety and blood product use and continuity of care domains. However, even in better-performing domains, there was room for improvement, with pretraining correct scores ranging around 60%.

**Table 1:** Pretraining and post-training assessment test scores (N=82) per domain

Domain (number of questions)	Pretraining*	Post-training*	Improvement†
General questions (5)	62.5%	90.8%	28.3
Infection control (3)	31.3%	86.7%	55.4
Medicine and blood use (3)	61.5%	83.7%	22.2
Surgery practice (1)	19.0%	76.0%	57.0
Birth practices (1)	17.8%	67.0%	49.2
Patient identification (2)	31.5%	60.7%	29.2
Communication skills (3)	28.2%	58.7%	30.5
Continuity of care (1)	58.3%	80.9%	22.6
Other adverse events (1)	22.6%	46.4%	23.8
Average	**37.0%**	**72.3%**	**35.3**

* Expressed as mean value of the percentage of correct answers per domain.

† Expressed as difference in percentage points between pretraining and post-training means.

After the training, test scores improved across all domains. The average post-training correct score rose to 72.3%—a substantial increase of over 35 percentage points. Improvement was observed in every patient safety domain assessed (table 1). The domains that had been weakest at baseline—surgery and birth practices—saw some of the largest gains. The percentage of correct answers on the surgical safety questions increased from a pretraining average of 19% to a post-training average of 76%, an absolute improvement of 57 percentage points. Similarly, obstetric safety knowledge rose from 17.8% to 67% correct on average (+49.2 percentage points). As noted, these domains were each measured with one question and results should be interpreted with caution. The infection prevention and control domain also showed a major improvement: correct responses went from 31.3% pretraining to 86.7% post-training, a gain of 55.4 percentage points. This domain included questions on topics such as hand hygiene indications, isolation precautions and appropriate antibiotic use—key issues highlighted by the situational analysis and targeted in the training. Each patient safety domain covered in the training showed a statistically significant improvement, with knowledge gains especially pronounced in areas that were initially identified as most deficient. These findings suggest that the training effectively addressed context-specific knowledge gaps, although further follow-up is needed to assess retention and application in practice.

## Discussion

The patient safety training programme described in this study was designed to cover the most pertinent patient safety knowledge areas for North Macedonia. The aim was to determine whether a short, intensive and context-tailored programme could measurably improve immediate knowledge of patient safety principles among healthcare workers, with the ultimate goal of informing a potential scale-up into a continuous, national-level professional development programme on patient safety.

Our findings clearly demonstrate that such a tailored training approach can lead to significant improvements in immediate patient safety knowledge among participants. Before the training, the baseline knowledge assessment revealed unsatisfactory levels in several critical patient safety. The large gains in post-training test scores indicate that the programme was effective in conveying key concepts and practices. Participants showed the largest improvements in areas that were previously weak, such as surgical safety, infection control and safe childbirth practices. However, domains assessed by single test items should be interpreted cautiously. It is reasonable to expect that improved awareness and immediate knowledge may act as a precursor to behaviour change and improved patient safety clinical practice, although our study did not directly measure long-term retention or clinical outcomes.

Based on informal feedback and discussions during the workshop, the training format—a combination of lectures and interactive group work—was well received by participants. Such mixed-method educational approach aligns with recommendations suggesting that adult learners benefit from active learning and contextual discussion.^20 21^ The post-training results lend support to the idea that engaging training methods, such as case discussions and problem-solving in groups, can enhance learning outcomes. These peer-based exchanges also helped foster a sense of shared responsibility and enabled the emergence of potential local ‘quality and safety champions’.

Other studies have similarly found that educational interventions can positively influence patient safety knowledge and culture, leading to better attitudes and behaviours regarding safety.^22 23^ A culture of learning in patient safety can be developed through continuous awareness-building and training supported by management and peers.^24^ Training programmes for hospital staff, focusing on teamwork, error management and patient involvement and delivered, via blended learning, were found to be feasible and potentially beneficial.^25^ In primary care settings, such educational interventions were found to significantly improve safety culture metrics and incident reporting rates among family medicine units.^26^ This supports the notion that training healthcare workers, in hospital and primary care settings, is a viable strategy to improve patient safety, complementing other system-level efforts.^27^

Our results reinforce and add to this body of evidence by providing data from an SEE country context that shows how a relatively short, context-tailored training programme can yield meaningful immediate knowledge gains. The training content was aligned with practical patient safety challenges in North Macedonia, which may have contributed to participant engagement. While the improvement in immediate knowledge is encouraging, knowledge alone does not equate to changes in practice. The ultimate goal of such training is to contribute to reducing patient harm and improving outcomes.^28^ This requires that healthcare workers not only know what to do but also consistently apply their knowledge and skills in their work environment. We did not measure practice change or patient outcomes in this study. Follow-up efforts, including onsite mentoring or refresher trainings, could help reinforce the application of what was learnt.

### Limitations

This study has several limitations. First, the evaluation relied on a short, 20-item multiple-choice questionnaire, which provides only a limited snapshot of participants’ knowledge. Some domains, such as surgical safety and safe childbirth practices, were each assessed with a single item, limiting the reliability of domain-specific conclusions. Second, the study did not include a control group, and there was no follow-up assessment to evaluate knowledge retention over time. Third, sampling approach and the relatively small sample size, as well as the exclusion of three participants who did not complete the post-test, may have introduced self-selection bias. Fourth, results were not stratified by profession or role, as sample sizes were insufficient to support subgroup analysis. Fifth, the study measured immediate knowledge gain only and did not assess behaviour change, implementation of practices or patient outcomes—limiting conclusions about longer term or system-level impact. Sixth, the 1-day training provided to managers was not included in the evaluation and is, therefore, not represented in the results. Finally, a limitation worth mentioning is that the participant sample was skewed towards secondary care, specifically hospitals and included fewer primary care workers. This was intentional to some extent, given many identified safety issues took place in hospitals. However, this means that the programme’s content and results may not fully apply to primary care or other settings. Future training efforts should strive to include a broader range of roles or, alternatively, develop separate training modules for primary care.

Several aspects of this training initiative and the accompanying research are worth highlighting. This was the first national-level patient safety training of its kind in North Macedonia, filling a gap in the country’s continuing education offerings. The training used a multimethod approach—combining theory and practice—and was delivered by a team of both international and local experts, lending credibility and a mix of perspectives. The content was directly aligned with identified system gaps, likely making it more impactful. These strengths point to the potential for replication: similar trainings could be adapted for other countries with comparable health system profiles. The issues of blame culture, underdeveloped incident reporting and inconsistent protocol adherence are not unique to North Macedonia; thus, a model of situational analysis followed by tailored training could be beneficial elsewhere.

Despite limitations, this training approach has provided proof-of-concept that a focused patient safety educational intervention can be delivered feasibly in North Macedonia and can produce rapid improvements in immediate knowledge. Future efforts should explore mechanisms for sustaining and scaling the intervention. These might include offering the training as a regular part of continuing professional development, embedding it into accreditation or licensing systems or developing complementary models such as mentorship schemes and peer learning circles. Longitudinal evaluation at 6–12 months could help assess retention and behavioural impact. While informal feedback from participants was positive, future editions should consider formal evaluation of participant satisfaction and suggestions for improvement.

This work contributes to a gradually growing evidence base on patient safety education.^2^ While such programmes generally seem to be effective, there is still a paucity of studies and a need for more robust evaluations. Our study adds data from a region that is under-represented in the literature. Continued sharing of lessons and results from different contexts will help build the case for prioritising patient safety training and inform best practices. 

## Conclusions

This study suggests that conducting a thorough situational analysis to identify context-specific gaps, followed by a tailored training programme targeting those gaps, can be beneficial towards improving patient safety at the healthcare system level. Although the gains observed were limited to immediate post-training knowledge, they highlight potential feasibility of delivering high-impact training to a diverse group of healthcare workers. This proof-of-concept effort also contributed to creating a space for engagement, reflection and dialogue: elements that are essential for promoting a positive safety culture. Strengthening health worker capacity in patient safety through such targeted training interventions can be a high-impact strategy for reducing preventable harm and improving the overall quality of care. Future efforts should prioritise the integration of patient safety into ongoing professional development. Institutionalising such training as part of accreditation or licensing processes could help scale and sustain these gains. In North Macedonia, and in other countries with similar challenges, embedding such training into a sustained, structured national strategy, including longer term evaluation and follow-up, may help reinforce learning and support lasting improvements in patient safety.

## Supplementary material

10.1136/bmjoq-2025-003473 online supplemental file 1

## Data Availability

Data are available upon reasonable request.
