# Supplementary material for: Developing and evaluating a proof-of-concept patient safety training programme for health workers in North Macedonia
Source: BMJ Open Qual. 2025 Sep 22;14(3):e003473. doi: 10.1136/bmjoq-2025-003473 (PMC12458890; doi:10.1136/bmjoq-2025-003473)
Supplement: online supplemental file 1 [file bmjoq-14-3-s001.pdf]

SUPPLEMENTARY MATERIALS

- Supplement 1: Training agenda
- Supplement 2: List of case scenarios used during groupwork
- Supplement 3: Assessment test

Supplement 1: Training agenda

Leadership and patient safety management in healthcare institutions in North Macedonia	
**Level 1 training: Workshop for the managers of healthcare institutions**	
Participants: Hospital managers	
AGENDA	
**Day 1: 14 November 2023**	
09:30–10:00	Opening
10:00–10:15	Quick online survey
10:15–10:45	Module 1:<br>Leadership and management for patient safety and strategies for including managers, clinical leaders, patients and their families in the care process
10:45–11:15	Module 2:<br>Undesirable events and the legal framework on patient safety at the national level and the role of Quality Improvement Commissions in the improvement of patient safety
11:15–11:30	Discussion and feedback from participants
11:30–12:00	Coffee break
12:00–12:15	Module 3:<br>Systemic approach, design, risk management strategy (the role of the manager in the creation of a culture of safety)
12:15–12:30	Module 4:<br>Measuring and improving the institution's performance (developing a Risk Management Action Plan at the local level)
12:30–12:45	Module 5:<br>Integrating patient safety training in formal and informal education programmes
12:45–13:15	Discussion and feedback from participants
13:15–13:30	Closing address
13:30–14:30	Lunch

Measures and activities for improving patient safety in healthcare institutions in North Macedonia	
**Level 2 training: Training programme for quality and clinical staff**	
Participants: Members of the Quality Committees within healthcare institutions	
AGENDA	
**Day 1: 15 November 2023**	
09:30–10:00	Opening
10:00–10:15	Pre-training test
10:15–10:45	<u>Module 1</u><br>Leadership for patient safety
10:45–11:15	Adverse events and legal framework for patient safety at the national level
11:15–11:30	Coffee break
11:30–12:00	<u>Module 2</u><br>Global challenge to patient safety: medicines without harm
12:00–12:30	Safe use of blood and blood products
12:30–14:30	Group work – case scenarios of different adverse events
14:30–15:30	Lunch

15:30–16:00	Group reporting
16:00–16:30	Questions and answers session
**Day 2: 16 November 2023**	
10:00–10:15	Review of Day 1
10:15–11:15	Module 3<br>Infection prevention and control
11:15–11:30	Coffee break
11:30–12:30	Group work
12:30–13:15	Group reporting
13:00–13:30	Module 4<br>Patient identification and communication<br>Patient safety across continuum of care
13:30–14:30	Group work
14:30–15:30	Lunch
15:30–16:00	Group reporting
16:00–16:30	Q&A session
**Day 3: 17 November 2023**	
09:30–10:00	Review of Day 2
10:00–10:30	Module 6<br>Safe surgery and birth<br>Other adverse events
10:30–11:30	Group work
11:30–11:45	Coffee break
11:45–12:45	Group reporting
12:45–13:15	Instructions for development of local action plans at the facility level
13:15–13:30	Q&A session
13:30–13:45	Post-training test
13:45–14:15	Lessons learned – recommendations and further steps
14:15–14:30	Closing remarks
14:30–15:30	Lunch

Supplement 2: List of case scenarios used during groupwork

List of case scenarios
(Four group works and nine scenarios provided each session)

Group work 1
**Adverse drug reactions and adverse events in blood use**
1. Prescription of look-alike-sound-alike medicines in internal department
2. Wrong prescription of anti-hypertension medicine
3. Unintentionally applied insulin instead of vaccine in three children
4. Unintentionally given epinephrine instead of midazolam for colonoscopy
5. Applied salbutamol in 10 times higher dose in a child (confused with decimals)
6. Confused calculation of amp. gentamycin due to two labels on the box
7. In elderly care home, error made in prescribing medicines within the file
8. Adverse reaction on blood transfusion due to extended time during transport
9. Wrong prescription of antibiotic in paediatric patient
Group work 2
**Hospital-acquired infections**
1. Adverse event following orthopaedics surgery
2. Hospital-acquired infection related to urinary catheter
3. Readmission related to surgery in appendectomy
4. Hepatitis B infections after discharge from department for internal diseases
5. Hospital-acquired infection with MRSA following abdominal surgery
6. Patient in ICU with ventilator-associated pneumonia with *Acinetobacter*
7. Detection of infections in patients operated in the same operation theatre
8. Skin infections in paediatric department
9. Infections in patients in dialysis department
Group work 3
**Patient identification, communication, and patient safety across continuum of care**
1. Identification of the newborn during the discharge procedure
2. Omitted identification in pharmacy while picking up prescription medications
3. Omitted identification of admitted patient in hospital before application of infusion
4. Error in identification in twins immediately after the birth
5. Inadequate communication with patient in intensive care unit
6. Transfer of an elderly patient from home to hospital and back to home
7. Transport of a patient from home to the nursing home
8. Patient admitted to the hospital as urgent case, transported by emergency ambulance
9. Hand over between shifts
Group work 4
**Adverse events in surgery, in birth practices, and other adverse events**
1. Delayed and inappropriate neonatal resuscitation
2. Birth of triplets in the delivery room around midnight
3. Delayed admission of a pregnant woman after three examinations on the same day
4. Delayed indication for caesarean section, and delivery of asphyxiated newborn
5. Neonatal resuscitation applied by inexperienced midwife
6. Neglected pregnant woman with labour pains
7. Fall of a patient in patient’s room (unrecognized risk of falls)
8. Suicide of a patient during the hospital stay
9. Broken tube with blood during transportation to the laboratory

**Questions to be answered in managing adverse events**<br>(Nationally accepted forms for management of adverse events were provided)
1. What was the adverse event in the case scenario?
2. In which step of the process did the event occur?
3. What was wrongly done? (root cause analysis)
4. What are the factors contributing to the adverse event? (root-cause analysis)
5. Should this case be reported? To whom?
6. Please complete the provided form for adverse event reporting.
7. Is this event preventable?
8. What are the preventive measures which could help in preventing such an event in the future?

Supplement 3: Assessment test

Assessment test

Outline of patient safety – General knowledge

Profile: A) Doctor B) Nurse/midwife C) Other profile

1. In the health-care institution, who must manage reported adverse events?
   a. manager (director)
   b. committee for quality-of-care improvement
   c. chief of department

2. What types of errors could happen?
   a. omitted actions
   b. wrong actions
   c. personal mistakes

3. Lack of time and overcrowded departments are:
   a. a systemic problem
   b. an individual barrier

4. Who must report the adverse event?
   a. patient
   b. health-care worker
   c. parent of patient, other family member, or guardian

5. Errors in treatment with medicines could happen during:
   a. prescription
   b. administration
   c. monitoring
   d. preparation
   e. dispensing
   f. all of the above

6. The most frequent and fatal hospital-acquired infections are:
   a. catheter-associated urinary tract infections
   b. central line-associated blood stream infections
   c. ventilator-associated pneumonia
   d. surgical site infections
   e. infections related to ultrasound examinations.

7. Risk factors for the transmission of hospital-acquired infections include:
   a. patient age
   b. antimicrobial resistance
   c. diagnostic/therapeutic methods
   d. sensitivity due to the current disease
   e. over-crowded unit (low staff/patient ratio)
   f. transfusions/infusions

8. In health-care institutions, who must report the adverse event?
   a. chief nurse
   b. the worker who noticed first, or who made an error
   c. committee for quality improvement

9. Acceptable modalities for patient identification are:
   a. name and surname
   b. date of birth

c. number of admission file
d. room and/or bed number
e. phone number
f. barcoding of admission information.

10. Communication is important for patient safety improvement in order to:
    a. prevent an adverse event
    b. manage an adverse event
    c. exchange information for facility events among staff
    d. exchange documents during transition of care

11. Fields where communication with patients is crucial include:
    a. informed consent
    b. patient admission
    c. medicines administration
    d. shift/duty handover
    e. preparation for surgery
    f. checklist for safe surgery
    g. conducting unit rounds

12. Types of wrong medication errors are:
    a. wrong choice of drug
    b. wrong dose of medicine
    c. wrong frequency
    d. already known allergy on the medicine prescribed
    e. allergy was provoked by prescribed medicine

13. Individual factors for non-efficient communication are:
    a. language barrier
    b. interpersonal conflict
    c. culture
    d. lack of knowledge
    e. noise

14. Hospital-acquired infections could be transmitted via:
    a. droplets
    b. contact
    c. food/drink
    d. vectors

15. Adverse drug reactions should be reported:
    a. electronically, on the website of the Agency for Drugs and Medical Devices
    b. in paper-based format to the Ministry of Health
    c. in paper-based format to the emergency department
    d. in paper-based format to the Agency for drug and medical devices

16. Where are the barriers during continuum of care within the health sector?
    a. systemic level
    b. individual level
    c. level of health-care workers
    d. other

17. Additional labelling of the patient should be performed for:
    a. food allergies
    b. confused patients
    c. patients with malignancies
    d. special diet due to disease
    e. patient with heart disease

18. Sentinel events (which should never happen) are:
    a. homicide
    b. suicide
    c. late visit to the patient
    d. surgery at wrong site
    e. dealing with soiled material
    f. wrong patient
    g. foreign object left inside body

19. In obstetrics and perinatology, adverse events which could be prevented are:
    a. pregnancy and intra partum infections
    b. inadequate birth position and loss of sense
    c. diagnostic errors
    d. breast feeding problems
    e. birth of placenta
    f. insufficient breast milk

20. Pressure ulcers (decubitus) are found mainly on:
    a. belly
    b. behind ears
    c. elbows
    d. hips
    e. back of head
    f. shoulders
    g. buttocks
    h. backs and sides of knees
